# Unraveling the volatile profile and bioactivity of Zhoupigan (*Citrus reticulata* cv. Manau Gan) essential oil via HS-GC-IMS and HS-SPME-GC-MS: a comprehensive evaluation of different extraction techniques

**DOI:** 10.3389/fnut.2026.1775469

**Published:** 2026-03-03

**Authors:** Huanhuan Xu, Jialiang Zou, Xuelian Gan, Fu Wang, Lin Chen, Hongping Chen, Yuan Hu, Youping Liu

**Affiliations:** 1 School of Pharmacy, Chengdu University of Traditional Chinese Medicine, Chengdu, China; 2 Chinese Medicine Germplasm Resources Innovation and Effective Uses Key Laboratory of Sichuan Province, Chengdu, China

**Keywords:** Bioactivity, Essential oils, HS-GC-IMS, HS-SPME-GC-MS, Zhoupigan (*Citrus reticulata* cv. Manau Gan)

## Abstract

**Introduction:**

Zhoupigan (*Citrus reticulata* cv. Manau Gan), also known as Zangju or Shitougan, is a distinctive Chinese citrus resource valued for both traditional medicinal uses and food flavoring, and is notably rich in essential oils (EOs). However, the chemical composition and bioactivity of Zhoupigan peel essential oil (ZJPEO) remain insufficiently understood, limiting its effective utilization.

**Methods:**

In this study, ZJPEO was extracted by steam distillation (SD) and supercritical fluid extraction (SFE) and analyzed using FTIR, E-nose, HS-GC-IMS and HS-SPME-GC-MS. And its antioxidant and antimicrobial activities were evaluated *in vitro*.

**Results:**

FTIR and E-nose revealed clear compositional differences between steam-distilled essential oil (SDEO) and supercritical fluid-extracted essential oil (SFEO). HS-GC-IMS detected 63 VOCs and, based on VIP > 1, identified five marker compounds. HS-SPME-GC-MS identified 1,250 VOCs, showing terpenoids and esters as major contributors to aroma and bioactivity. KEGG enrichment highlighted sesquiterpene and triterpene biosynthesis as key pathways. Flavor wheel analysis indicated that SFEO had stronger sweet, green, and woody notes and exhibited potent antioxidant and antimicrobial activities associated with specific VOCs.

**Discussion:**

Overall, this work supports optimizing ZJPEO extraction and promoting its value-added, sustainable utilization.

## Highlights

- Comprehensive chemical characterization of ZJPEO by HS-GC-IMS and HS-SPME-GC-MS.
- SFE extraction yielded higher levels of bioactive compounds than SD.
- SFEO demonstrated superior antioxidant and antimicrobial activities.
- Key differential VOCs (e.g., thymol) correlated with bioactivities were identified.
- Extraction methods significantly affect the chemical profile, flavor and bioactivity of ZJPEO.

## 1 Introduction

Citrus is one of the most extensively cultivated fruits worldwide, valued for its unique flavor and nutritional benefits. Major citrus varieties include mandarin (*Citrus reticulata*), grapefruit (*C. paradisi*), lemon (*C. limon*) and lime (*C. aurantifolia*) (1). The rapid expansion of the citrus processing industry has generated considerable quantities of peel waste, which poses growing environmental concerns (2). Nevertheless, citrus peels are rich in citrus essential oils (CEOs), which have attracted increasing interest due to their distinctive aroma, diverse bioactivities, and low toxicity (3). Zhoupigan (*C. reticulata* cv. Manau Gan), also known as Zangju or Shitougan, is a unique Chinese citrus variety with a cultivation history spanning thousands of years. Characterized by its wrinkled peel, tender and juicy flesh, and sweet-sour taste with a slight bitterness, Zhoupigan is widely recognized for its traditional medicinal property of “clears heat without causing internal fire.” It is primarily cultivated in high-altitude regions such as Derong and Muli in Sichuan (commonly known as Zangju), where warm climates, large diurnal temperature differences, and fertile soils contribute to its distinctive flavor profile (4). In addition, it is also cultivated in Ankang, Shaanxi (commonly known as Shitougan) (5) and Huarong, Hunan (commonly known as Huarong Zhoupigan) (6). Zhoupigan peel is an important source of CEOs, which are dominated by terpenes and oxygenated terpenoids such as aldehydes, esters and alcohols. Key aromatic and bioactive constituents include *D*-limonene, β-pinene, and linalool identified (7). Increasing evidence indicates that volatile organic compounds (VOCs) in CEOs possess antimicrobial, antioxidant and anti-inflammatory activities, suggesting their great potential as natural preservatives (8). Owing to their characteristic aroma and functional properties, CEOs are widely used in perfumery, cosmetics and as flavor enhancers in food products including beverages and ice cream (9). Despite Zhoupigan peel's distinctive flavor and abundant essential oils (EOs), the absence of systematic research has constrained its processing, industrial development and broader utilization.

Driven by increasing demand for the efficient use of natural plant resources, the development of high-quality and value-added EOs products has become a research hotspot (10). Extraction represents a critical step in unlocking the bioactive potential of plant-derived EOs for industrial, pharmaceutical and therapeutic applications. The extraction method plays a decisive role in shaping the chemical composition and biological properties of EOs (11). Currently, steam distillation (SD) are used to extract more than 90% of EOs, owing to their simple equipment requirements and their ability to yield natural, contamination-free products (12). However, prolonged heating during SD can degrade thermolabile components, consequently altering the oil's natural aroma profile (13). To address these limitations, supercritical fluid extraction (SFE) has gained widespread use as a green and efficient modern technique. It is widely applied in the extraction of spices and traditional Chinese medicinal materials. Operating at low temperatures with high efficiency, SFE effectively prevents the degradation of heat-sensitive constituents. Nonetheless, supercritical CO_2_ is a low-polarity solvent. Consequently, it exhibits limited capability in extracting polar compounds. This results in extracts that differ from those produced by traditional methods (12). Substantial evidence confirms that extraction methods profoundly influence EOs composition, thereby affecting their sensory attributes and bioactivities. For example, Park et al. (14) reported that cold pressing (CP) extracted specific VOCs such as sesquiterpenes, oxygenated monoterpenes and fatty acid derivatives, whereas SD yielded distinct compounds like terpinyl cation derivatives, limonene and 4-vinylguaiacol. Zeng et al. (15) reported that 3-methylbutyraldehyde was the key flavor compound in *Camellia oleifera* EOs obtained via CP, hot pressing (HP) and microwave-assisted extraction (MAE), while soxhlet extraction (SE) predominantly produced ethyl acetate. Furthermore, Zhu et al. (16) demonstrated that SD-extracted *Semen Platycladi* EOs exhibited superior antioxidant and antimicrobial properties compared with extracts obtained by heated reflux extraction (HRE) and ethanol soxhlet extraction (ESE). Similarly, Yan et al. (17) reported that SFE significantly enhanced chromone content and improved antioxidant and anti-inflammatory activities in agarwood oil relative to SD extraction. Collectively, these findings indicate that extraction techniques substantially influence the composition, aroma and bioactivities of EOs. However, how different extraction methods affect Zhoupigan peel essential oils (ZJPEO) remains largely unexplored. This knowledge gap restricts its potential for industrial-scale processing and high-value product development. Thus, investigating the effects of SD and SFE on ZJPEO is essential for advancing the development of high-quality, value-added ZJPEO products.

Currently, analytical methods used for characterizing VOCs in EOs include both sensory evaluation and instrumental analysis. Sensory analysis, though informative, is subjective and heavily dependent on evaluator experience, making it difficult to quantify. In contrast, instrumental approaches offer objective and precise detection at the molecular level (18). Fourier transform infrared spectroscopy (FTIR) enables the identification of functional groups and major chemical constituents through characteristic absorption bands, providing a rapid and reliable tool for EOs quality assessment and preliminary compositional analysis (19). Recent studies have further confirmed the practical application of FTIR in the authentication of EOs. For example, Manin et al. (75) successfully achieved effective differentiation of natural bergamot EOs from adulterated samples by identifying characteristic alcohol absorption peaks using FTIR-ATR combined with pattern recognition techniques such as PCA and random forest model. The electronic nose (E-nose), incorporating sensor arrays and pattern recognition algorithms to mimic the human olfactory system, reduces sensory subjectivity and provides holistic information about aroma profiles. However, it lacks the capability to identify or quantify specific compounds (20). Headspace solid-phase microextraction gas chromatography-mass spectrometry (HS-SPME-GC-MS) is widely applied for VOCs analysis due to its high separation efficiency and strong identification and quantification performance, although its sensitivity for trace compounds may be limited (21). Fortunately, headspace gas chromatography-ion mobility spectrometry (HS-GC-IMS) compensates for this limitation. As a powerful technique for detecting trace volatiles based on ion drift time differences, HS-GC-IMS excels at analyzing VOCs present at low concentrations and offers advantages such as rapid analysis, minimal sample preparation, high resolution, intuitive visualization and atmospheric-pressure operation (22, 23). Nonetheless, its database remains incomplete, leaving some VOCs unidentified (24). Given the limitations of any single method, combining multiple analytical techniques enables more comprehensive VOC profiling. This approach facilitates the detection of a broader range of compounds and yields more detailed flavor characteristics, representing an increasingly prevalent research trend (25, 26).

In this study, the effects of steam distillation (SD) and supercritical fluid extraction (SFE) on the chemical composition and bioactivity of Zhoupigan peel essential oil (ZJPEO) were systematically investigated. The chemical profiles were characterized using FTIR, E-nose, HS-GC-IMS and HS-SPME-GC-MS, while antioxidant and antimicrobial activities were evaluated in parallel. The results provide a scientific basis for optimizing ZJPEO extraction processes, highlight its potential for developing high-value ZJPEO products and promote the sustainable utilization and deeper industrial exploitation of Zhoupigan resources.

## 2 Materials and methods

### 2.1 Materials and reagents

Zhoupigan was harvested in December 2023 from Derong County, Ganzi Tibetan Autonomous Region, Sichuan Province, China (99° 16′ 37″ E; 28° 32′ 32″ N; 2,225 meters), and healthy fruits of uniform maturity, similar size, and free from mechanical damage and pests and diseases were selected at the time of harvesting. Zangju peel (ZJP) samples were obtained after the peel was obtained by manual separation and dried in the sun under natural conditions. Subsequently, the raw samples were randomly divided into two batches and used for the extraction of EOs by different extraction methods (steam distillation and supercritical fluid extraction).

Hexane was purchased from Merck & Co., Inc. (Rahway, NJ, USA), and it was chromatographically pure. Sodium chloride was purchased from Sinopharm (Beijing, China). Ethyl alcohol, potassium persulfate, sodium acetate anhydrous, ferrous sulfate, iron (III) chloride, tween 80 and DMSO were all purchased from Chengdu Kelong Chemical Co., Ltd. (Chengdu, Sichuan Province, China). 1,1-diphenyl-2-picrylhydrazyl (DPPH), 22′-azino-bis (3-ethylbenzothiazoline-6-sulfonic acid) diammonium salt (ABTS), TPTZ and gentamicin sulfate were all purchased from Shanghai Macklin Biochemical Co., Ltd. (Shanghai, China). Ascorbic acid (Vc) was purchased from Aladdin Biochemical Technology Co., Ltd. (Shanghai, China). TTC stain solution was purchased from Shanghai Yuanye Bio-Technology Co., Ltd. (Shanghai, China). LB nutrient agar medium and LB broth medium were purchased from Qingdao Hi-Tech Park Haibo Biotechnology Co., Ltd. (Qingdao, Shandong Province, China). All the above reagents were analytically pure. Potassium bromide was purchased from Chengdu Kelong Chemical Co., Ltd. (Chengdu, Sichuan Province, China) and it was spectral pure. *Staphylococcus aureus* (ATCC6538) and *Escherichia coli* (ATCC25922) were both purchased from Beijing Biological Depository Center (Beijing, China).

### 2.2 Preparation of ZJPEO

The ZJP essential oil (ZJPEO) in Section 2.1 were obtained by steam distillation (SD) and supercritical fluid extraction (SFE). The steam-distilled essential oil (SDEO) was provided by Sichuan Tianfu Aromatherapy Health Technology Research Institute Co., Ltd. (Sichuan, China), with a yield of 2.7%.

The supercritical fluid-extracted essential oil (SFEO) was provided by Nantong Hua'an Supercritical Extraction Co., Ltd. (Jiangsu, China). The specific extraction conditions were as follows: the sample was placed in a supercritical CO_2_ extraction apparatus (HA220-50-06, Nantong Hua'an Supercritical Extraction Co., Ltd., Jiangsu, China). The extraction pressure was set at 30 MPa, extraction temperature at 42 °C, CO_2_ flow rate at 22 L·h^−1^, pressure of separation vessel 1 at 10 MPa, temperature of separation vessel 1 at 56 °C, pressure of separation vessel 2 at 6 MPa, temperature of separation vessel 2 at 47 °C, and extraction time of 2 h. After extraction, the extract was collected from the separation vessels to obtain SFEO, with a yield of 2.2%. The received EOs were stored in amber glass bottles protected from light and kept in a refrigerator at 4 °C for further use.

### 2.3 FTIR analysis

The functional groups of SDEO and SFEO were characterized using a FTIR spectrometer. An appropriate amount of potassium bromide was placed in an agate mortar and ground finely. It was then pressed under a pressure of 15 kN for 10 s to form a transparent thin slice. After background scanning of the blank slice, approximately 25 μL of the sample was dropped onto the blank slice to form a uniform thin layer, and its infrared spectrum was measured. The instrument conditions were as follows: the spectral range was from 4,000 cm^−1^ to 400 cm^−1^, the number of scans was 32, and the resolution was 4 cm^−1^.

### 2.4 E-nose analysis

Twenty microliters of ZJPEO was placed in a 4 mL headspace injection vial, sealed and equilibrated at 25 °C for 30 min, an E-nose instrument (Isensortalk Co., Ltd., Beijing, China) was employed. The detection time was 60 s, and the cleaning time was 120 s. The analytes were analyzed in a 4 mL headspace injection vial.

### 2.5 HS-GC-IMS analysis

HS-GC-IMS was performed to analyze the VOCs of the two ZJPEOs using a GC-IMS instrument (FlavourSpec^®^, G.A.S., Dortmund, Germany) equipped with a headspace autosampler (CTC Analytics AG, Zwingen, Switzerland). Ten microliters of ZJPEO was diluted to 1 mL in a 20 mL headspace vial and incubated at 60 °C for 15 min, then C4-C9 n-alkanone (Shanghai Aladdin Biochemical Technology Co., Ltd., Shanghai, China) were used as external standards. The VOCs were identified according to the drift times of the standards in the RI and GC-IMS libraries (G.A.S.). The carrier gas for GC was nitrogen (purity ≥ 99.999%). The carrier gas flow rate was 2 mL/min in 2 min, 10 mL/min in 2–10 min, and 100 mL/min in 10–20 min. IMS conditions: the IMS temperature was 45 °C, the drift gas flow rate the was 150 mL/min, and the drift gas was nitrogen (purity ≥ 99.999%).

### 2.6 HS-SPME-GC-MS analysis

The chemical composition of ZJPEO was analyzed by Agilent 8890-7000D GC-MS/MS (Agilent J&W Scientific, Folsom, CA, USA) according to the method of Yuan et al. (27). Take 10 μL of ZJPEO, add 9,990 μL of n-hexane, 100 μL was injected into the headspace vial after vortex mixing, and then 20 μL (10 μg/mL) of internal standard solution was added. For SPME analysis, the headspace vial was shaken at a constant temperature of 60 °C for 5 min, and then a 120 μm DVB/CWR/PDMS extraction head (Agilent J&W Scientific, Folsom, CA, USA) was inserted into the sample headspace vial, headspace extraction was performed for 15 min, and desorption was carried out at 250 °C for 5 min. The sample was analyzed by using an Agilent 8890-7000D gas chromatography-mass spectrometry instrument equipped with a DB-5MS capillary column (30 m × 0.25 mm × 0.25 μm, Agilent J&W Scientific, Folsom, CA, USA) with high-purity helium (purity ≥ 99.999%) as the carrier gas, and an inlet temperature of 250 °C. The extraction was carried out at a constant flow rate of 1.2 mL/min. The programmed temperature increase: 40 °C was held for 3.5 min, 10 °C/min to 100 °C, 7 °C/min to 180 °C, and 25 °C/min to 280 °C for 5 min.

The mass spectra were recorded in 70 eV electron impact (EI) ionization mode. The temperatures of the quadrupole mass detector, ion source and mass spectrometry interface were set at 150, 230, and 280 °C, respectively. The mass spectra were performed in selected ion monitoring (SIM) mode for qualitative and quantitative analysis of MS data.

Compound identification was conducted based on an in-house database, MWDB (MetWare Biological Co., Ltd., Wuhan, Hebei Province, China). First, the mass spectrometry data were processed using MassHunter software with the following deconvolution parameters: peak width set to 20, resolution, sensitivity, and chromatographic peak shape all set to medium, and a minimum match factor of 70. The acquired mass spectra were compared against the NIST (2020) mass spectral library for preliminary compound identification. To enhance the accuracy of identification, we established a retention index (RI) filtering criterion: only matches with a difference of ≤ 30 units between the calculated RI and the reference RI in the NIST library were retained (the retention index was calculated using a homologous series of n-alkanes, C_7_–C_40_, as standards). Subsequently, through comprehensive comparison of retention indices, actual components, and characteristic ions, the chemical structures and names of the volatile compounds corresponding to each chromatographic peak were ultimately confirmed (28). For quantification, one quantitative ion and two to three qualitative ions were selected for each compound. Integration and calibration were performed using the quantitative ions, and the relative content of each component was determined using the peak area normalization method.

The relative odor activity value (rOAV) analysis was performed according to the method of Huang et al. (29). This method, used to identify key flavor compounds, calculates the contribution of individual volatiles to a sample's overall flavor profile based on their concentration and sensory thresholds. Typically, an rOAV ≥ 1 indicates that a compound makes a direct contribution to the flavor.

### 2.7 Biological activity evaluation

#### 2.7.1 Evaluation of antioxidant activity

The DPPH and ABTS free radical scavenging abilities and the FRAP total antioxidant capacity of ZJPEO at different concentrations (0.5–10 mg/mL) were determined with reference to the method of Flieger and Flieger (30); Priyanthi and Sivakanesan (31) with slight modifications. The DPPH and ABTS free radical scavenging abilities were expressed as the half-inhibitory concentration (IC_50_) and the FRAP total antioxidant capacity was expressed as mmol/g. The standard curve was *y* = 0.7309 x + 0.0366 (*R*^2^ = 0.9991).

#### 2.7.2 Evaluation of antibacterial activity

##### 2.7.2.1 Detection of diameter of inhibition zone (DIZ)

The Oxford cup method (32) was used to determine the antibacterial effects of ZJPEO against *Staphylococcus aureus (S. aureus)* and *Escherichia coli (E. coli)*. One hundred microliters of bacterial suspension at a concentration of 10^6^ CFU/mL was added to the medium and 100 μL of the ZJPEO stock solution was added to an Oxford cup placed on the surface of the medium and incubated in an incubator at 37 °C for 24 h. The antimicrobial activity of the ZJPEO was assessed by measuring the DIZ around the Oxford cup, which was recorded as millimeters (mm). Sterile water was used as negative control and gentamicin sulfate as positive control.

##### 2.7.2.2 Determinations of minimal inhibitory (MIC) and bactericidal concentration (MBC)

The micro broth dilution method of Ksouda et al. (33) was used to determine the MIC and MBC of ZJPEO against bacteria with slight modifications. LB broth was used to prepare a series of concentrations of ZJPEO (512–1 μL/mL) twofold dilutions of ZJPEO (containing 1% DMSO and 1% Tween 80). One hundred microliters of each ZJPEO dilution was taken and 20 μL of bacterial solution (10^6^ CFU/mL) was added to each well and incubated at 37 °C for 24 h. Ten microliters of TTC staining solution (20 mg/mL) was added to each well and stained for 30 min at 37 °C. The color change of each well was observed and the minimum ZJPEO concentration without color development was taken as the MIC. To estimate the MBC, 20 μL of the staining solution (20 mg/mL) was taken from the wells with no significant bacterial To estimate the MBC, 20 μL was removed from the wells with no obvious bacterial growth, applied to LB agar plates, incubated at 37 °C for 24 h, removed, observed and counted and the MBC was taken as the number of ≤ 5 colonies.

### 2.8 Data analysis

The results presented in the statistical analysis tables and figures represent the mean values from three independent experiments, expressed as mean ± standard deviation (SD). For GC-IMS, the VOCal software was used to view the spectrograms and quantify the data and the NIST and IMS databases built into the VOCal software were used for qualitative analysis of VOCs. For GC-MS, the MassHunter software was used for process the data, conduct qualitative and quantitative analyses and integrate and correct the chromatographic peaks. The peak area of each chromatographic peak represents the relative content of the corresponding VOCs.

Principal component analysis (PCA), cluster heatmap analysis, classification analysis, flavor wheel analysis, and correlation analysis were performed using the publicly available online platform Metware Cloud (https://cloud.metware.cn). Prior to modeling, the data underwent log_2_ transformation and UV (unit variance scaling). Subsequently, the MetaboAnalystR package in R was employed to construct an orthogonal partial least squares-discriminant analysis (OPLS-DA) model. The validity of the model was assessed through 200 permutation tests, with the following acceptance criteria: the intercept of the Q^2^ regression line from the permutation test must be less than 0.05 (or at least negative), and the Q^2^ value of the original model must be greater than 0.5 (or meet the commonly used threshold in the field), thereby ensuring reliable predictive capability. Variable importance in projection (VIP) scores were extracted from the validated OPLS-DA model and combined with fold-change (FC) analysis (screening criteria: FC ≥ 2 or FC ≤ 0.5 and VIP > 1) to identify differential VOCs. In addition, SPSS 25.0 (IBM Corp., Armonk, NY, USA) was used for statistical analysis (with *P*<*0.05* indicating statistical significance), and GraphPad Prism 10.1.2 (GraphPad Software, San Diego, CA, USA) was utilized to generate bar charts. Metabolite annotation was conducted based on the KEGG database, covering both compounds (https://www.kegg.jp/kegg/compound/) and metabolic pathways (http://www.kegg.jp/kegg/pathway.html).

## 3 Results and discussion

### 3.1 FTIR analysis

**Figure 1:**
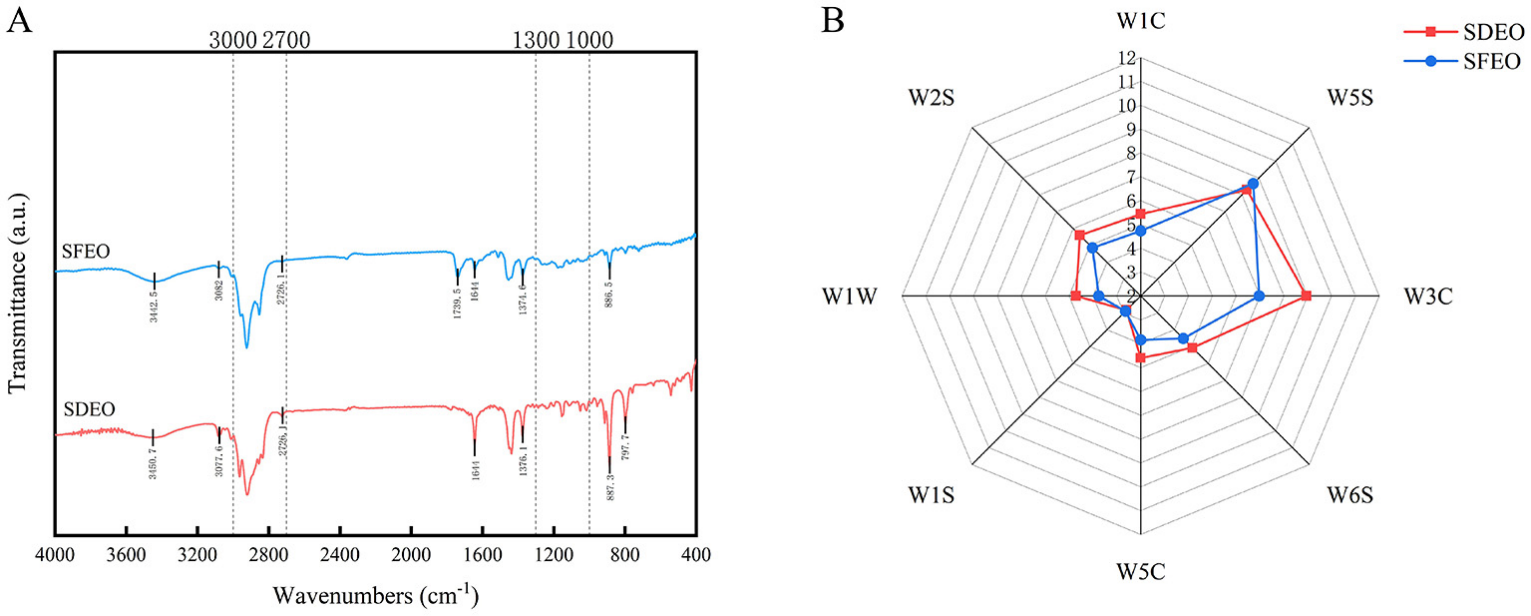
ZJPEO FTIR spectrum and E-nose analysis of different extraction methods. **(A)** FTIR spectrum. **(B)** E-nose radargram.

As shown in Figure 1A, the infrared spectra of ZJPEO extracted by the two methods (SDEO and SFEO) are similar. Both spectrafeature multiple absorption peaks corresponding to characteristic molecular vibrations. The major functional groups present in both extracts are consistent. Broad absorption bands around 3,442–3,451 cm^−1^ are assigned to O-H stretching vibrations (34), indicating that ZJPEO contains abundant alcohols. The stronger O-H signal in the SFEO sample suggests that this technique may extract volatile alcohols more efficiently. The peaks at 3,082, 3,077.6, and 797.7 cm^−1^ correspond to = C-H stretching and out-of-plane C-H bending in terpenoids. Another peak in the spectrum appears at 1,644 cm^−1^, corresponding to the stretching vibration of the C=C bond in terpenoids (35). Since extraction methods can alter the efficiency of terpenoid recovery, the higher peak intensity observed in SDEO implies a greater terpenoid content. Terpenoids are abundant in CEOs, with limonene (a hydrocarbon monoterpene) being the most abundant compound (36). These terpenoids contribute to the anti-inflammatory, antibacterial and other bioactivities of CEOs (37, 38). Stretching vibrations of C-H bonds in alkanes are observed in the 3,000–2,700 cm^−1^ region. Additionally, bending vibrations at 1,374.6 and 1,376.1 cm^−1^ further confirm the presence of alkanes (39). Additional C-H bending at 2,726.1 cm^−1^, together with the C=O stretching peak at 1,739.5 cm^−1^, indicates the presence of aldehydes (40). Bands in the 1,000–1,300 cm^−1^ region are indicative of C-O stretching, suggesting that most compounds in ZJPEO are oxygenated terpenoids, wihich are typically aromatic (41). Additionally, C-H bending vibrations in aromatic compounds are observed at 886.5 and 887.3 cm^−1^ (40). Collectively, these spectral features indicate that ZJPEO comprises alcohols, terpenoids, alkanes and aldehydes. Although the overall spectral profiles of SDEO and SFEO are broadly similar, subtle differences exist in the intensity and shape of several absorption peaks. These variations point differences in the concentrations of specific constituents. These qualitative findings suggest that the extraction method influences the chemical composition of the ZJPEO, warranting further confirmation through quantitative analysis.

### 3.2 E-nose analysis

The E-nose sensor array exhibited distinct response patterns to VOCs present in ZJPEO (Supplementary Table S1). Among them, the W5S (sensitive to nitrogen oxides) and W3C (sensitive to ammonia) sensors showed particularly strong responses (Figure 1B), indicating relatively high levels of nitrogen oxides and ammonia-containing aromatic compounds. Notably, this observation differs from the findings reported by Wang et al. (42), which may be attributed to differences in sample physical state or sensor sensitivity. With the exception of the W5S sensor, SDEO generally elicited stronger responses than SFEO, suggesting that the extraction method markedly affects the overall aroma characteristics of the ZJPEO. PCA (Supplementary Figure S1) showed that the two ZJPEO samples were clearly separated into distinct clusters, confirming significant compositional differences and divergent odor profiles. However, the E-nose is inherently insensitive to certain key aroma-active substances in ZJPEO (42). Thus, it cannot capture the complete chemical signature nor provide qualitative or quantitative identification of individual VOCs. Instead, it offers only an overview of the detectable portion of the volatile profile. Taken together, these results reveal notable differences in VOC compositions between the ZJPEO samples extracted by SD and SFE. Therefore, more precise analytical techniques are required for their accurate identification and comprehensive comparison.

### 3.3 HS-GC-IMS analysis

#### 3.3.1 HS-GC-IMS spectral analysis

**Figure 2:**
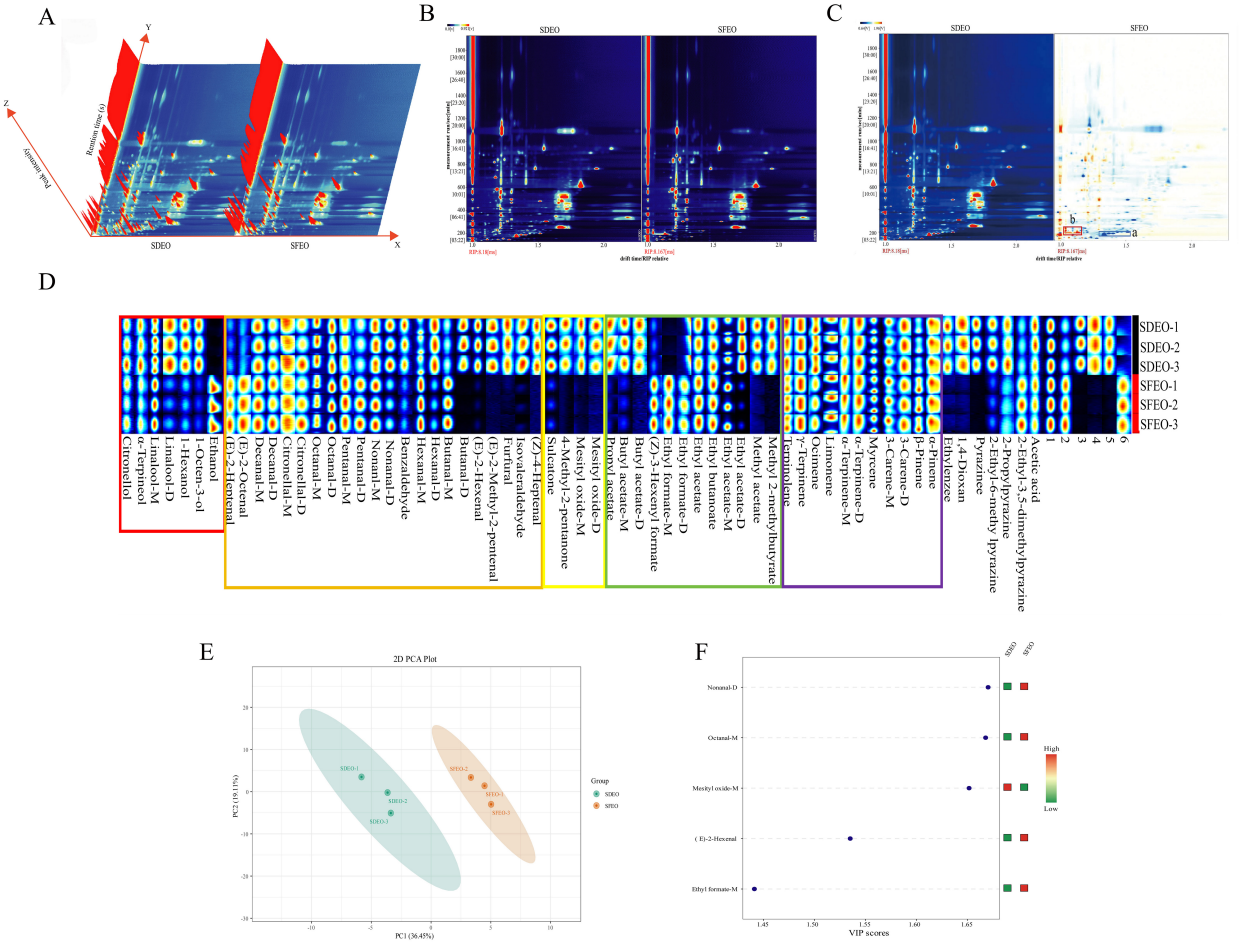
VOCs spectra and key VOCs analysis of ZJPEO with different extraction methods. **(A)** Three-dimensional spectrogram. **(B)** Two-dimensional spectrogram. **(C)** Difference comparison diagram. **(D)** Fingerprint diagram. **(E)** PCA analysis result graph. **(F)** VIP score graph.

HS-GC-IMS is an emerging analytical technique that enables rapid, highly sensitive detection of VOCs, offering strong separation capability and intuitive visualization of aroma characteristics (43). In the three-dimensional HS-GC-IMS spectrum obtained in this study (Figure 2A), the x-axis, y-axis, and z-axis represent drift time, retention time and signal intensity, respectively. The overall spectral patterns of SDEO and SFEO appeared similar, suggesting comparable VOC compositions; however, variations in peak intensities indicate differences in their relative abundance. Because the three-dimensional spectrum is comparatively coarse, the data were further processed into a two-dimensional top-view plot to facilitate more detailed comparison (Figure 2B). In this plot, the background is blue and a red vertical line at an abscissa value of 1.0 corresponds to the reaction ion peak (RIP). Each signal point denotes a specific VOC, with its color intensity reflecting concentration: white indicates low abundance, red represents high abundance, and darker shades correspond to progressively higher levels. Most VOCs in both samples eluted within a retention time range of 100–1,200 s and a drift time of 1.0–2.0 ms, with those eluting between 100 and 600 s showing higher density, while VOCs between 600 and 1,200 s were more dispersed. This variation is likely due to differences in compound polarity, causing polar and non-polar constituents to exhibit distinct retention times when interacting with the non-polar stationary phase of the chromatographic column (44).

To visualize the differences in VOCs between extraction methods, a two-dimensional GC-IMS difference spectrum was generated (Figure 2C). Here, SDEO was set as the reference and the spectra of other samples were subtracted from it. In the resulting plot, the blue background indicates signals similar to the reference, red denotes higher signal intensity and blue signifies lower intensity relative to the reference. Color depth corresponds to the magnitude of difference. Regions marked blue (a) indicate lower abundance than SDEO, whereas red regions (b) indicate higher abundance. For more detailed comparison, these data can be further transformed into a fingerprint profile.

#### 3.3.2 Identification and analysis of VOCs by HS-GC-IMS

The results of the VOCs identification and analysis for ZJPEO are presented in Table 1. A total of 69 signal peaks were detected by HS-GC-IMS. By comparing drift times and retention indices against standards, 63 VOCs were identified; the remaining peaks could not be identified due to database limitations, underscoring the need for an expanded HS-GC-IMS database for more comprehensive VOC profiling (24). Notably, some compounds produced multiple signal peaks (e.g., monomers, dimers, trimers), which is attributed to variations in VOCs concentration (45). As shown in Supplementary Figure S2A, the VOCs of SDEO and SFEO were broadly similar, encompassing 22 aldehydes (34.92%), 12 esters (19.05%), 11 terpenoids (17.46%), 7 alcohols (11.11%), 7 other compounds (11.11%), and 4 ketones (6.35%), with a greater variety of compounds detected compared to the FTIR results. As shown in Table 1, the major VOCs of SDEO were ethyl butanoate, 2-methyl methylbutyrate, (*E*)-2-octenal, mesityl oxide-*M* and 3-carene-*M*. In contrast, the major VOCs of SFEO were ethanol, 2-propylpyrazine, octanal-*M*, butanal-*D*, and ocimene. The ethanol content in SFEO (4.49%) was significantly higher than that in SDEO (1.11%). According to literature reports, after SFE extraction, the pipeline system and valves of the SFE equipment must be cleaned using solvents (e.g., ethanol, water, or mixtures thereof), which may lead to partial retention of ethanol in the final product (46). This may explain the higher ethanol content observed in SFEO. In the study by Song et al. (47), ethanol levels as high as 9.17%−11.89% were detected by GC-IMS. In contrast, although the ethanol content in SFEO in the present study is higher than that in SDEO, it remains within the commonly reported range. Moreover, high ethanol concentrations can generate strong background signals in E-nose detection, thereby reducing its ability to discriminate odors. It has also been reported that the presence of other components can attenuate the influence of ethanol on the E-nose response (48). Therefore, the overall odor discrimination capability of the E-nose results from the combined effects of multiple volatile constituents. Aldehydes were abundantly extracted in both SDEO (31%) and SFEO (36.83%), with SFEO showing a comparatively higher proportion of aldehyde compounds. Ketone content was significantly lower in SFEO compared with SDEO (*P* < 0.05*;* Supplementary Figure S2B). No significant differences were observed among other compound classes, likely because the high temperature during SD may cause degradation of thermolabile compounds (16). In addition, 13 monomeric compounds exhibited significantly different abundances between extraction methods (Supplementary Figure S2C), confirming that extraction parameters influence EOs composition and may consequently alter biological activities. For example: linalool-*M* was more abundant in SDEO (1.24 times that of SFEO), suggesting greater potential for anticancer and anxiolytic applications (49). In contrast, citronellal-*D* was more prevalent in SFEO (1.61 times that of SDEO), indicating stronger antimicrobial and repellent functionality, making SFEO more suitable for natural antimicrobial/repellent product development (49, 50). Overall, the extraction method can be strategically selected to modulate ZJPEO composition and optimize its functional properties.

**Table 1:** HS-GC-IMS identification of volatile components of ZJPEO by different extraction methods.

**Count**	**Compound**	**CAS**	**Formula**	**MW**	**RI**	**Rt (s)**	**Dt (ms)**	**Relative content(%)**	
								**SDEO**	**SFEO**
Alcohol								7.78 ± 0.03^a^	9.87 ± 0.05^a^
1	α-Terpineol	98-55-5	C_10_H_18_O	154.3	1,724.8	1,575.633	1,30504	1.25 ± 0.02^a^	1.06 ± 0.01^a^
2	Linalool-M	78-70-6	C_10_H_18_O	154.3	1,566.7	1,097.705	1.22551	1.54 ± 0.00^a^	1.24 ± 0.00^b^
3	Linalool-D	78-70-6	C_10_H_18_O	154.3	1,565.6	1,094.928	1.76592	1.35 ± 0.02^a^	0.45 ± 0.01^a^
4	Ethanol	64-17-5	C_2_H_6_O	46.1	952.9	194.755	1.13237	1.11 ± 0.01^a^	4.49 ± 0.03^a^
5	1-Octen-3-ol	3391-86-4	C_8_H_16_O	128.2	1,441.2	824.016	1.17206	1.38 ± 0.00^a^	0.6 ± 0.01^a^
6	1-Hexanol	111-27-3	C_6_H_14_O	102.2	1,380.7	717.589	1.33093	0.45 ± 0.01^a^	0.84 ± 0.00^a^
7	Citronellol	106-22-9	C_10_H_20_O	156.3	1789	1824521	1.36869	0.7 ± 0.01^a^	1.19 ± 0.01^a^
Aldehyde								31.00 ± 0.05^a^	36.83 ± 0.02^a^
8	Decanal-M	112-31-2	C_10_H_20_O	156.3	1,500.2	942.904	1.54494	0.41 ± 0.00^a^	1.52 ± 0.01^a^
9	Decanal-D	112-31-2	C_10_H_20_O	156.3	1,500.2	942.904	20.5473	2.28 ± 0.02^a^	1.37 ± 0.02^a^
10	Benzaldehyde	100-52-7	C_7_H_6_O	106.1	1,512	968.721	1.15431	1.80 ± 0.00^a^	1.45 ± 0.01^a^
11	Citronellal-M	106-23-0	C_10_H_18_O	154.3	1,481.2	902.997	1.35776	0.63 ± 0.00^a^	0.63 ± 0.00^a^
12	Citronellal-D	106-23-0	C_10_H_18_O	154.3	1,481.6	903.695	1.89786	0.84 ± 0.00^b^	1.35 ± 0.00^a^
13	Nonanl-M	124-19-6	C_9_H_18_O	142.2	1,410.4	767.949	1.48189	1.14 ± 0.00^b^	1.94 ± 0.00^a^
14	Nonanal-D	124-19-6	C_9_H_18_O	142.2	1,408.8	765.133	1.94363	0.40 ± 0.00^b^	0.82 ± 0.00^a^
15	Octanal-M	124-13-0	C_8_H_16_O	128.2	1,315.2	617.883	1.41106	0.94 ± 0.00^b^	3.17 ± 0.00^a^
16	Octanal-D	124-13-0	C_8_H_16_O	128.2	1,311.4	612.433	1.82083	1.59 ± 0.01^a^	2.08 ± 0.00^a^
17	Pentanal-M	110-62-3	C_5_H_10_O	86.1	1,004.9	224.219	1.19389	1.56 ± 0.01^a^	1.19 ± 0.00^a^
18	Pentanal-D	110-62-3	C_5_H_10_O	86.1	1,005.7	224.761	1.42179	1.56 ± 0.00^a^	0.99 ± 0.01^a^
19	Furfural	98-01-1	C_5_H_4_O_2_	96.1	1,473.1	886.344	1.08695	1.05 ± 0.00^a^	1.71 ± 0.01^a^
20	(E)-2-Heptenal	18829-55-5	C_7_H_12_O	112.2	1,331.1	640.677	1.26651	0.82 ± 0.00^a^	0.80 ± 0.00^a^
21	(E)-2-Octenal	2548-87-0	C_8_H_14_O	126.2	1,441	823.614	1.33756	3.39 ± 0.03^a^	1.91 ± 0.02^a^
22	(E)-2-Hexenal	6728-26-3	C_6_H_10_O	98.1	1,235.5	481.881	1.51787	1.32 ± 0.00^b^	2.76 ± 0.00^a^
23	Hexanal-M	66-25-1	C_6_H_12_O	100.2	1,105.1	305.69	1.26896	1.11 ± 0.01^a^	1.66 ± 0.01^a^
24	Hexanal-D	66-25-1	C_6_H_12_O	100.2	1,105.4	306.106	1.55859	2.28 ± 0.03^a^	0.64 ± 0.00^a^
25	(E)-2-Methyl-2-pentenal	14250-96-5	C_6_H_10_O	98.1	1,161.4	373.008	1.50839	0.78 ± 0.00^a^	2.33 ± 0.03^a^
26	Isovaleraldehyde	590-86-3	C_5_H_10_O	86.1	941.9	189.223	1.4062	1.32 ± 0.01^a^	0.97 ± 0.01^a^
27	Butanal-M	123-72-8	C_4_H_8_O	72.1	850.5	148.748	1.11593	2.35 ± 0.02^a^	2.06 ± 0.02^a^
28	Butanal-D	123-72-8	C_4_H_8_O	72.1	847.4	147.55	1.28215	1.90 ± 0.01^a^	3.02 ± 0.00^a^
29	(Z)-4-Heptenal	6728-31-0	C_7_H_12_O	112.2	1,229.5	472.128	1.13658	1.52 ± 0.01^a^	2.51 ± 0.02^a^
Terpenoids								20.27 ± 0.03^a^	17.76 ± 0.03^a^
30	γ-Terpinene	99-85-4	C_10_H_16_	136.2	1,256.6	517.668	1.70651	1.54 ± 0.00^b^	1.74 ± 0.00^a^
31	Terpinolene	586-62-9	C_10_H_16_	136.2	1,281.1	562.743	1.29555	1.91 ± 0.00^a^	1.07 ± 0.01^a^
32	Ocimene	13877-91-3	C_10_H_16_	136.2	1,236.8	483.932	1.68689	1.83 ± 0.01^a^	3.02 ± 0.00^a^
33	Limonene	138-86-3	C_10_H_16_	136.2	1,213.8	447.547	1.6575	2.59 ± 0.00^a^	2.58 ± 0.00^a^
34	α-Terpinene-M	99-86-5	C_10_H_16_	136.2	1,195	419.74	1.21614	1.16 ± 0.00^a^	1.08 ± 0.01^a^
35	α-Terpinene-D	99-86-5	C_10_H_16_	136.2	1,192.7	416.521	1.7258	2.38 ± 0.02^a^	0.98 ± 0.00^a^
36	Myrcene	123-35-3	C_10_H_16_	136.2	1,179.4	397.388	1.28959	1.97 ± 0.01^a^	0.72 ± 0.00^a^
37	β-Pinene	127-91-3	C_10_H_16_	136.2	1,121.5	323.974	1.29985	0.40 ± 0.00^a^	1.91 ± 0.02^a^
38	3-Carene-M	13466-78-9	C_10_H_16_	136.2	1,140.8	346.829	1.63583	3.15 ± 0.00^a^	1.65 ± 0.02^a^
39	3-Carene-D	13466-78-9	C_10_H_16_	136.2	1,138.1	343.505	2.18806	2.29 ± 0.00^a^	2.01 ± 0.02^a^
40	α-Pinene	80-56-8	C_10_H_16_	136.2	1,046.9	254.945	1.67275	1.05 ± 0.01^a^	1.00 ± 0.00^a^
Ketone								6.98 ± 0.01^a^	3.42 ± 0.02^b^
41	Sulcatone	110-93-0	C_8_H_14_O	126.2	1,362.6	688.501	1.18009	0.80 ± 0.00^a^	0.86 ± 0.00^a^
42	Mesityl oxide-M	141-79-7	C_6_H_10_O	98.1	1,153	362.204	1.11835	3.30 ± 0.00^a^	0.49 ± 0.00^b^
43	Mesityl oxide-D	141-79-7	C_6_H_10_O	98.1	1,153.1	362.204	1.44467	2.05 ± 0.00^a^	1.62 ± 0.02^a^
44	4-Methyl-2-pentanone	108-10-1	C_6_H_12_O	100.2	1,020.9	235.457	1.48151	0.83 ± 0.01^a^	0.46 ± 0.00^a^
Ester								24.38 ± 0.05^a^	20.81 ± 0.07^a^
45	Ethyl acetate-M	141-78-6	C_4_H_8_O_2_	88.1	909.4	173.708	1.09797	2.05 ± 0.02^a^	1.10 ± 0.01^a^
46	Ethyl acetate-D	141-78-6	C_4_H_8_O_2_	88.1	908.5	173.301	1.33476	1.92 ± 0.02^a^	1.49 ± 0.01^a^
47	(Z)-3-Hexenyl formate	33467-73-1	C_7_H_12_O_2_	128.2	1,283.4	567.055	1.1646	1.00 ± 0.01^a^	1.81 ± 0.01^a^
48	Butyl acetate-M	123-86-4	C_6_H_12_O_2_	116.2	1,093.5	293.91	1.24154	0.99 ± 0.00^b^	1.49 ± 0.00^a^
49	Butyl acetate-D	123-86-4	C_6_H_12_O_2_	116.2	1,092.9	293.383	1.61833	1.67 ± 0.01^a^	2.53 ± 0.00^a^
50	Ethyl butanoate	105-54-4	C_6_H_12_O_2_	116.2	1,031.7	243.361	1.21642	4.74 ± 0.00^a^	2.64 ± 0.02^a^
51	Methyl 2-methylbutyrate	868-57-5	C_6_H_12_O_2_	116.2	1,018.3	233.582	1.53745	4.07 ± 0.03^a^	0.46 ± 0.00^a^
52	Propyl acetate	109-60-4	C_5_H_10_O_2_	102.1	993.4	216.713	1.16199	1.13 ± 0.00^a^	2.55 ± 0.02^a^
53	Ethyl acetate	141-78-6	C_4_H_8_O_2_	88.1	829.9	140.898	1.07116	2.30 ± 0.00^a^	1.17 ± 0.01^a^
54	Ethyl formate-M	109-94-4	C_3_H_6_O_2_	74.1	860.7	152.823	1.06932	0.65 ± 0.00^b^	2.03 ± 0.00^a^
55	Ethyl formate-D	109-94-4	C_3_H_6_O_2_	74.1	859.6	152.344	1.22327	2.98 ± 0.04^a^	1.48 ± 0.00^a^
56	Methyl acetate	79-20-9	C_3_H_6_O_2_	74.1	861.2	153.006	1.02933	0.89 ± 0.01^a^	2.05 ± 0.02^a^
Others								9.58 ± 0.01^a^	11.31 ± 0.01^a^
57	Acetic acid	64-19-7	C_2_H_4_O_2_	60.1	1,479.6	899.675	1.0524	2.23 ± 0.02^a^	0.58 ± 0.00^a^
58	2-Ethyl-6-methylpyrazine	13925-03-6	C_7_H_10_N_2_	122.2	1,402.6	754.458	1.19377	0.75 ± 0.01^a^	1.78 ± 0.00^a^
59	2-Ethyl-3,5-dimethylpyrazine	13925-07-0	C_8_H_12_N_2_	136.2	1,471.1	882.347	1.22378	1.19 ± 0.00^a^	0.84 ± 0.01^a^
60	2-Propylpyrazine	18138-03-9	C_7_H_10_N_2_	122.2	1,425.5	795.052	1.19275	1.09 ± 0.01^b^	3.72 ± 0.01^a^
61	Pyrazine	290-37-9	C_4_H_4_N_2_	80.1	1,200	426.981	1.04487	0.45 ± 0.01^b^	1.75 ± 0.00^a^
62	Ethylenzee	100-41-4	C_8_H_10_	106.2	1,170.1	384.643	1.07201	3.07 ± 0.04^a^	0.80 ± 0.00^a^
63	1,4-Dioxan	123-91-1	C_4_H_8_O_2_	88.1	1,088.8	289.715	1.12626	0.80 ± 0.01^b^	1.84 ± 0.00^a^

Different lowercase letters in the same row represent significant differences at *P* < 0.05; M, monomer; D, dimer; MW, molecular weight; RI, retention index; RT, retention time; Dt, drift time.

#### 3.3.3 HS-GC-IMS fingerprint analysis

To directly compare the VOC profiles of SDEO and SFEO, fingerprint plots were constructed using the Gallery Plot plugin (Figure 2D). In these fingerprint spectra, each row corresponds to a sample (including three biological replicates) and each column to a specific VOC, with darker colors representing higher relative abundance. The substance in the red box represents alcohols. Among them, linalool-*D*, 1-hexanol, and 1-octen-3-ol were more abundant in SDEO, whereas ethanol was more higher in SFEO. The substance in the orange box represents aldehydes. Among them, butanal-*D*, (*E*)-2-hexenal, (*E*)-2-methyl-2-pentenal, furfural, isovaleraldehyde, and (*Z*)-4-heptenal were present at higher levels in SDEO. The marked increase in (*E*)-2-hexenal may stem from polyunsaturated fatty acid degradation (51). The substance in the yellow box represents ketones, all ketones were substantially higher in SDEO, suggesting that SD may enhance their release and extraction. The substance in the green box represents esters. Propyl acetate, butyl acetate *M/D*, Methyl 2-methylbutyrate, and methyl acetate were enriched in SDEO, while (*Z*)-3-hexenyl formate and ethyl formate *M/D* were more abundant in SFEO. The substance in the purple box represents terpenoids. The two extraction methods showed no significant difference in terpenoid extraction efficiency, with minimal variation in content.

PCA revealed clear clustering between samples (Figure 2E), demonstrating that the two extraction methods generated distinct VOCs profiles. Using a VIP threshold of > 1, five key differential VOCs were identified: nonanal-*D*, octanal-*M*, mesityl oxide-*M*, (*E*)-2-hexenal, and ethyl formate-*M* (Figure 2F). These VOCs may serve as chemical markers for distinguishing ZJPEO obtained via different extraction methods. These findings provide a theoretical basis for establishing a scientific quality control and traceability system, thereby supporting standardized production, process optimization and the targeted development of high-value ZJPEO-based products.

### 3.4 HS-SPME-GC-MS analysis

#### 3.4.1 Analysis of VOCs in SDEO and SFEO

**Figure 3:**
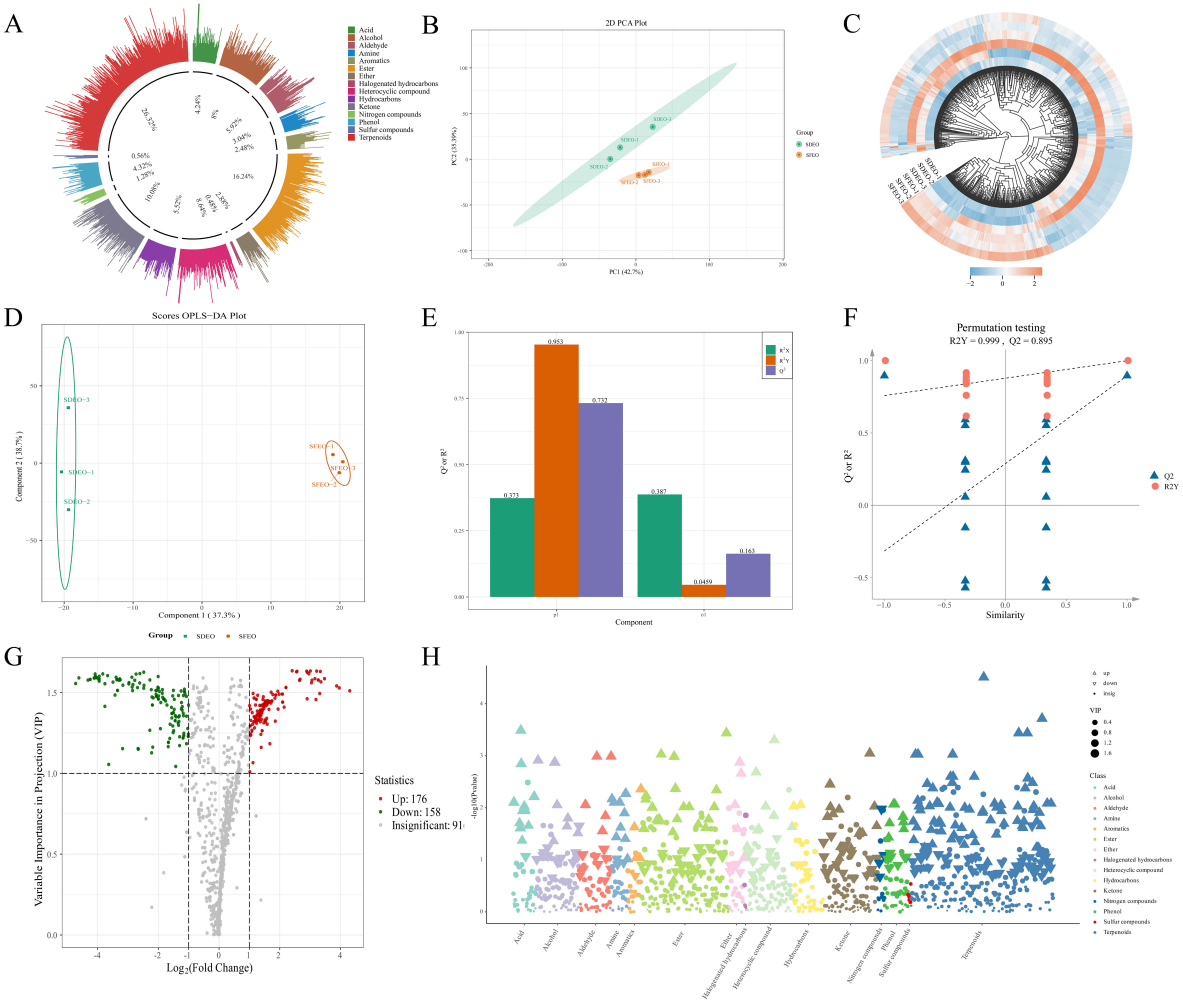
Differences between different extraction methods of ZJPEO and VOCs generalization. **(A)** Pie chart of categorization of identified VOCs. **(B)** Plot of PCA analysis results. **(C)** Heat map of VOCs clustering. **(D)** OPLS-DA score plot. **(E)** OPLS-DA model. **(F)** OPLS-DA alignment test. **(G)** Differential VOCs volcano plot. **(H)** Differential VOCs classification scatter plot. In the volcano plot, each dot represents a different substance, with the size of the dot indicating the magnitude of its corresponding VIP value. Different colors represent the expression levels of substances with significant differences after threshold screening. In the classification scatter plot, the x-axis represents metabolite categories, with each color representing a specific class of substances, and the y-axis represents the significance of differences. An upward-pointing triangle indicates upregulated substances, a downward-pointing triangle indicates downregulated substances, and a dot represents substances with no significant differences. The size of the triangle/circle indicates the magnitude of the VIP value.

HS-SPME-GC-MS was employed to comprehensively identify and characterize the VOCs in ZJPEO. As shown in Figure 3A, a total of 1,250 VOCs were detected, with the total ion chromatogram presented in Supplementary Figure S3. These compounds were categorized into 15 chemical classes (Supplementary Table S2), including 329 terpenoids (26.32%), 203 esters (16.24%), 126 ketones (10.08%), 108 heterocyclic compounds (8.64%), 100 alcohols (8%), 74 aldehydes (5.92%), 69 hydrocarbons (5.52%), 54 phenols (4.32%), 53 acids (4.24%), 38 amines (3.04%), 36 ethers (2.88%), 31 aromatic hydrocarbons (2.48%), 16 nitrogen-containing compounds (1.28%), 7 sulfur-containing compounds (0.56%), and 6 halogenated hydrocarbons (0.48%). HS-SPME-GC-MS analysis showed that terpenoids, esters, and ketones were the most diverse classes of compounds, comprising 658 species (52.64%). In contrast, HS-GC-IMS results indicated that aldehydes, esters, and terpenoids were the major contributors to the volatile aroma profile, accounting for 45 species (71.43%), with aldehydes alone representing 34.92%. The observed discrepancies may be attributed to the differing sensitivities of HS-GC-IMS and HS-SPME-GC-MS techniques in detecting VOCs. HS-GC-IMS exhibits higher sensitivity toward low-molecular-weight and low-boiling-point VOCs, whereas it shows limited response to high-boiling-point compounds (52, 53). When detecting high-boiling-point VOCs via GC-IMS, their low volatility often leads to adsorption within the chromatographic column or transfer lines, thereby compromising detection accuracy (54). In contrast, GC-MS demonstrates superior sensitivity toward high-molecular-weight and high-boiling-point VOCs. Furthermore, studies indicate that GC-MS can identify specific alkanes that remain undetected by GC-IMS due to their low proton affinity, which hampers efficient ionization in IMS (55). Methodologically, the two techniques also differ: GC-MS typically requires complex sample pretreatment procedures, while GC-IMS enables direct headspace sampling, simplifying preprocessing and allowing intuitive visualization of VOC profiles between samples through color-contour imaging (56). Consequently, reliance on a single analytical technique is insufficient for comprehensive VOC characterization. This underscores the necessity of employing complementary analytical approaches to achieve a more complete and accurate representation of the VOC composition in samples (57). Also upgrading and improving existing instrumental methods can enhance their sensitivity and accuracy in chemical composition detection. For example, a photoionization detector (PID) enhanced with a thin zeolite 5A layer can improve the selectivity for fatty acid markers (e.g., palmitic acid) in complex oil matrices, thus providing a fast and sensitive solution for the screening of key markers in complex matrices (58). In addition to this, the complementary sensor-based strategy of this study provides a proven solution to such problems. PCA demonstrated clear separation between SDEO and SFEO samples (Figure 3B), indicating substantial differences in their VOC profiles. This conclusion was reinforced by hierarchical clustering analysis, which highlighted marked variations in VOCs abundance between the two extraction methods (Figure 3C).

#### 3.4.2 Analysis of differential VOCs

**Figure 4:**
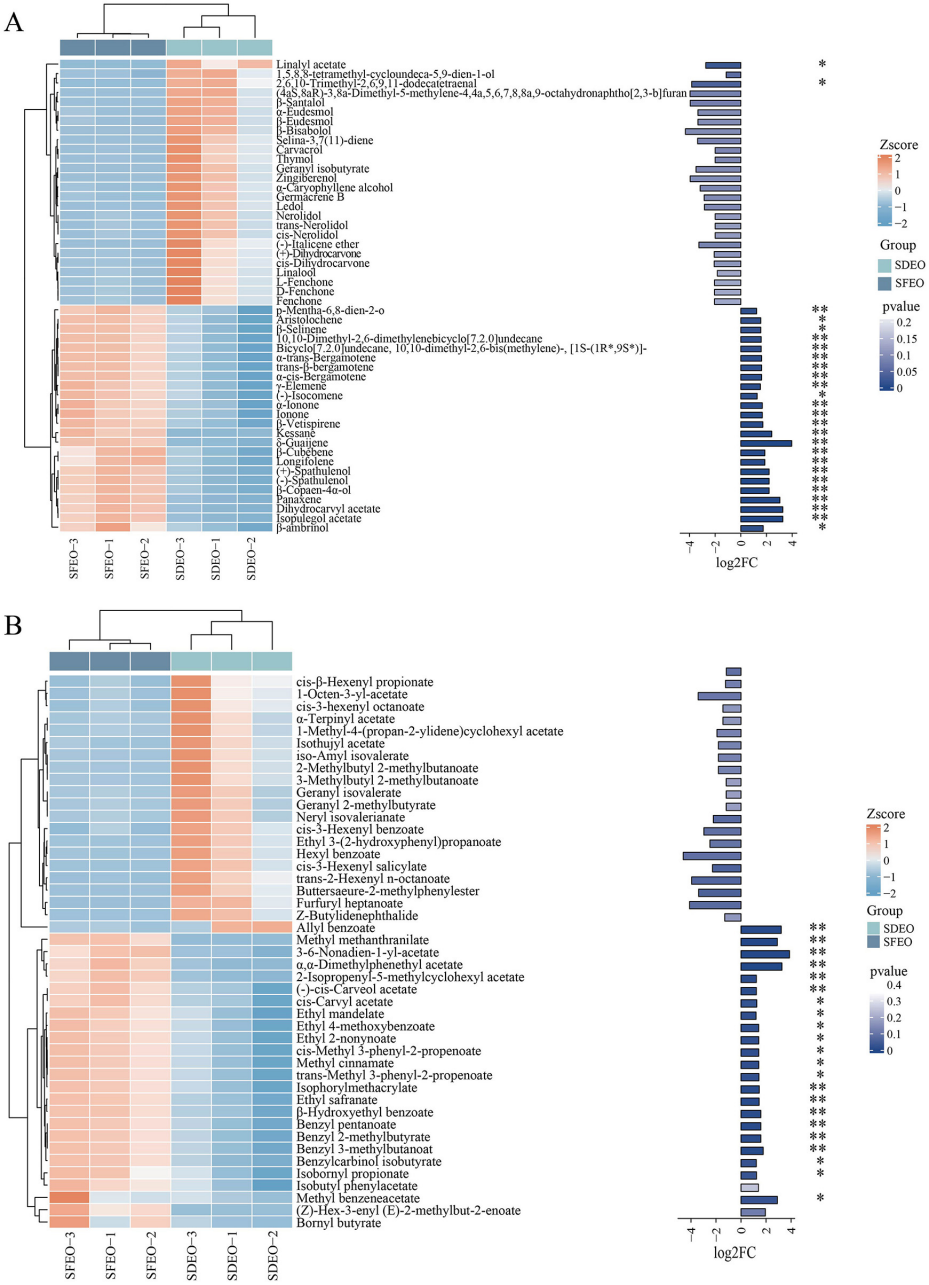
Cluster analysis of VOCs differing between extraction methods ZJPEO. **(A)** Terpenoids. **(B)** Esters. The length of the columns in the bar chart on the right represents the magnitude of the log2FC value; the color and * represent the *P*-value, **Indicates an extremely significant difference (*P* < 0.01), *Indicates a significant difference (*P* < 0.05).

To further investigate compositional differences between SDEO and SFEO, an OPLS-DA model was constructed. The score plot exhibited distinct separation between the two groups (Figure 3D), indicating significant metabolic divergence. A permutation test (*n* = 200) was conducted to validate the OPLS-DA model. With R^2^X (0.76), R^2^Y (0.999), and Q^2^ (0.895) all exceeding 0.5, the model demonstrated good reliability and fitting performance, making it suitable for subsequent differential VOCs screening analysis (Figures 3E, F). Using the filtering criteria of FC ≥ 2 or FC ≤ 0.5 and VIP > 1, a total of 334 differential VOCs were identified between SFEO and SDEO (176 upregulated, 158 downregulated) (Figure 3G). Terpenoids constituted the most abundant class among these differential VOCs, with 119 compounds (69 upregulated, 50 downregulated), followed by esters (45; 24 upregulated, 21 downregulated), ketones (34; 14 upregulated, 20 downregulated), and alcohols (26; 10 upregulated, 16 downregulated) (Figure 3H). Notable upregulated terpenoids included kessane, dihydrocarvyl acetate, isopulegol acetate, panaxene, and (-)-spathulenol, while major upregulated esters comprised methyl methanthranilate, (*Z*)-hex-3-enyl (*E*)-2-methylbut-2-enoate and 2-isopropenyl-5-methylcyclohexyl acetate. These results indicate that terpenoids and esters are the primary differential VOCs classes distinguishing SFEO from SDEO (Supplementary Table S3). To visualize this, a heatmap of the top 50 esters and terpenoids VOCs by VIP value (Figure 4) confirmed significant differences in their metabolic levels between the two samples, suggesting that these compounds likely contribute to the distinct aroma and bioactivity of ZJPEO.

**Figure 5:**
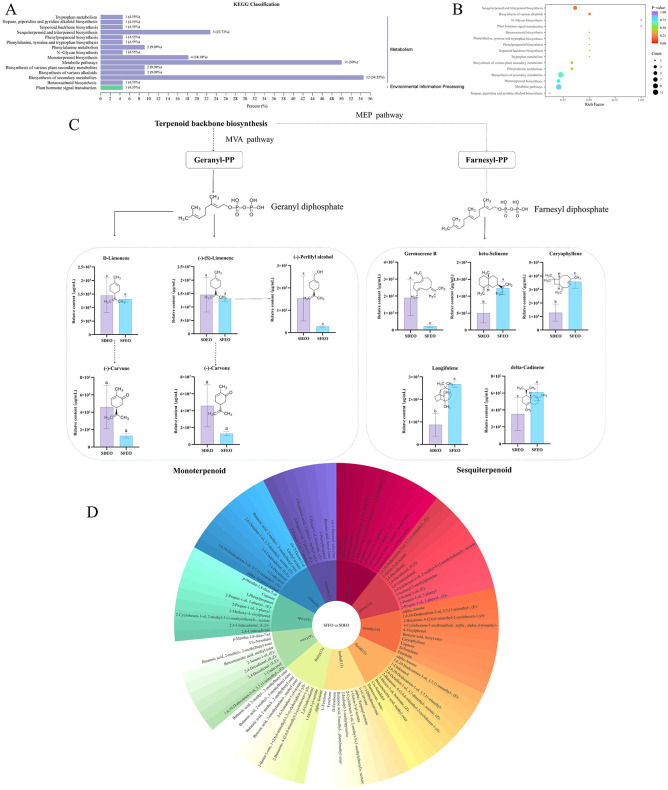
KEGG enrichment analysis and flavor analysis of ZJPEO differential VOCs by different extraction methods. KEGG enrichment analysis diagram. The x-axis indicates the enrichment factor, and the y-axis the pathways. The larger red dots represent the main pathway enrichment and higher pathway impact values, individually. Different lowercase letters indicate significant differences (*P* < *0.05*) in the agreement metrics. Solid lines indicate direct synthesis and dotted lines indicate synthesis through several steps. The second circle of the flavor analysis plot shows the number of metabolites annotated to different sensory flavors, and the outermost circle shows the differential VOCs. **(A)** SFEO vs. SDEO KEGG pathway annotation. **(B)** SFEO vs. SDEO KEGG enrichment analysis. **(C)** Differential metabolite terpenoid synthesis pathways. **(D)** SFEO vs. SDEO flavor wheel.

The KEGG database is a major public pathway repository used to investigate metabolite accumulation within biological networks (59). KEGG pathway annotation was subsequently performed to explore the biological pathways associated with differential VOCs accumulation. Most differential VOCs were associated with sesquiterpenoid and triterpenoid biosynthesis, as well as monoterpenoid biosynthesis (Figure 5A). This was supported by KEGG enrichment analysis, which showed significant enrichment of these VOCs specifically in sesquiterpenoids and triterpenoids biosynthesis pathways (Figure 5B). To elucidate the metabolic routes involved, a terpenoid biosynthesis map was constructed (Figure 5C). CEOs are rich in monoterpenes and sesquiterpenes, which accumulate in specialized oil glands of the peel and in the oil bodies of juice sacs (3). Within the terpenoid biosynthesis pathway, SFEO exhibited higher levels of key differential VOCs. These compounds originate from the terpenoid precursors farnesyl pyrophosphate (FPP) and geranyl pyrophosphate (GPP), supplied by the MVA and MEP pathways. These intermediates are converted by terpene synthases into metabolites such as *D*-carvone, (-)-carvone, germacrene *B*, β-selinene, caryophyllene, longifolene, and δ-cadinene, many of which possess anti-inflammatory, antibacterial, antioxidant, analgesic, and anticonvulsant activities (60–62).

#### 3.4.3 Flavor characterization of differential VOCs

Zhoupigan is highly appreciated for its distinctive flavor profile and nutritional properties. VOCs are key determinants of flavor quality and provide objective insight into sensory attributes. To assess the sensory contribution of differential VOCs, all 334 differential VOCs were annotated using established odor databases (https://www.odour.org.uk; https://www.flavornet.org; https://www.femaflavor.org/flavor-library). The 10 most frequently annotated flavor attributes (rOAV ≥ 1) were selected to construct a flavor wheel (Figure 5D). Compared with SDEO, SFEO exhibited a more diverse and intense flavor profile, showing stronger associations with sweet, green, woody, floral, herbal, fruity, waxy, spicy, citrus and balsamic notes. These findings suggest that the distinctive flavor of SFEO results from the combined contribution of its uniquely enriched differential VOCs.

### 3.5 Biological activity evaluation

#### 3.5.1 Antioxidant activity

**Figure 6:**
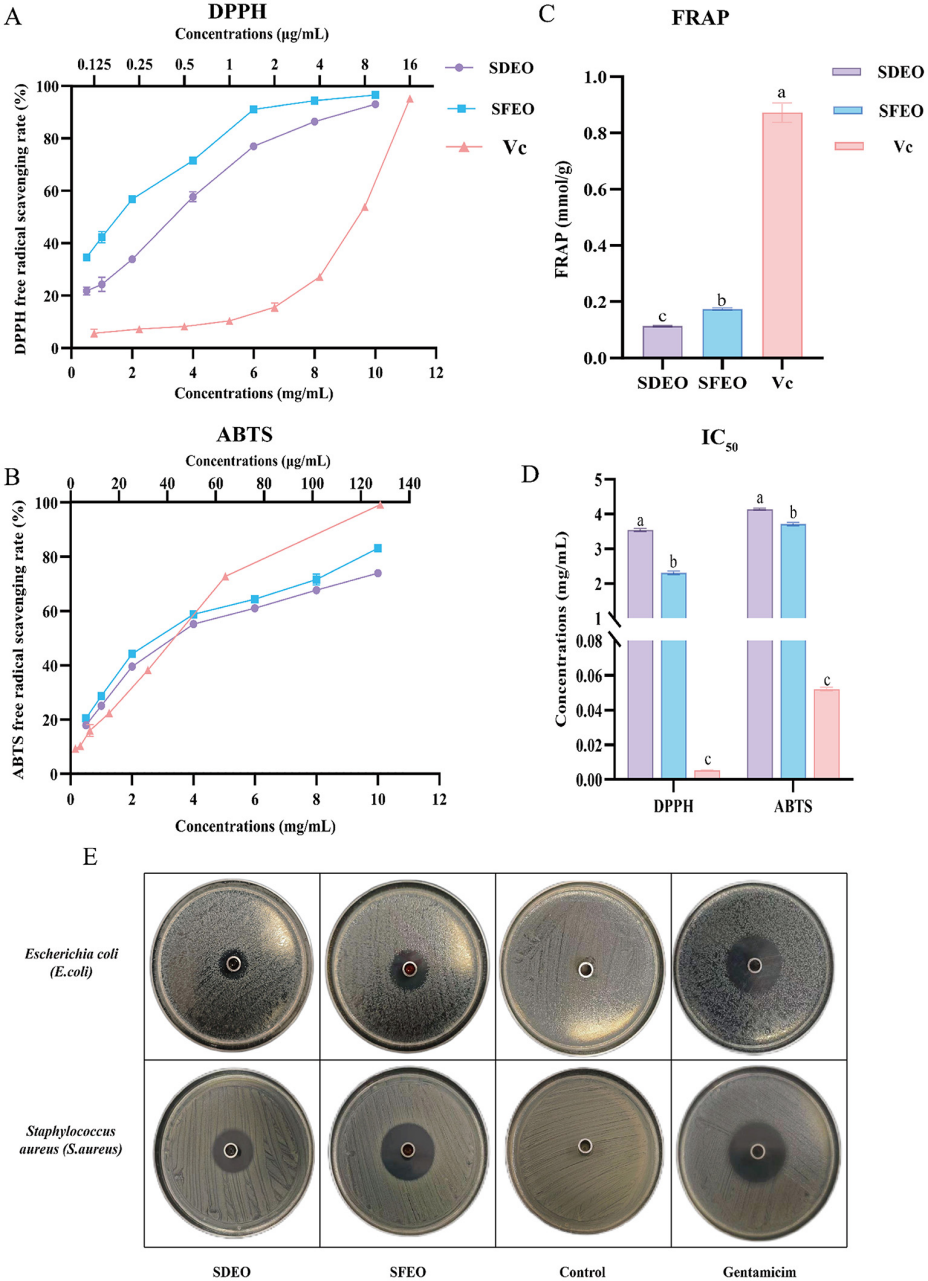
Comparison of bioactivities of ZJPEO extracted by different methods. **(A)** DPPH free radical scavenging ability. **(B)** ABTS free radical scavenging ability. **(C)** FRAP total antioxidant capacity. **(D)** IC_50_ values of DPPH and ABTS scavenging rates. **(E)** Circle of inhibition assay results. Different lowercase letters indicate significant differences in the same index (*P* < *0.05*).

CEOs are rich in natural antioxidants capable of mitigating oxidative stress and related disorders, making them promising alternatives to synthetic antioxidants in the food industry (63). In this study, the antioxidant activities of SFEO and SDEO were evaluated using DPPH, ABTS, and FRAP assays. Both types of ZJPEO displayed concentration-dependent scavenging activities against DPPH and ABTS radicals (Figures 6A, B). The total antioxidant capacity measured by the FRAP assay indicated that SFEO (FRAP = 0.1741 mmol/g) was significantly higher than that of SDEO (FRAP = 0.1135 mmol/g, *P*<*0.05*; Figure 6C). For DPPH radical scavenging activity, the IC50 of SFEO was 2.31 mg/mL, while that of SDEO was 3.54 mg/mL, and the IC50 of the positive control (vitamin C) was 5.45 μg/mL; SFEO exhibited stronger DPPH radical scavenging activity, which was significantly superior to that of SDEO (*P*<*0.05*), though both were lower than vitamin C. Similarly, the IC50 of SFEO for ABTS radical scavenging was 3.71 mg/mL, that of SDEO was 4.14 mg/mL, and that of the positive control (vitamin C) was 52.16 μg/mL; the ABTS radical scavenging activity of SFEO was also significantly higher than that of SDEO (*P*<*0.05*; Figure 6D). Overall, SFEO consistently demonstrated superior antioxidant activity compared to SDEO. We speculate that prolonged exposure to elevated temperatures during steam distillation may induce the oxidation or degradation of thermolabile constituents, subsequently reducing the antioxidant capacity of SDEO (64). The antioxidant properties of CEOs are known to depend on both the abundance and composition of bioactive constituents (9), with oxygenated monoterpenes contributing substantially to enhanced antioxidant potential (65). The present findings confirm that ZJPEO possesses notable antioxidant activity and represents a promising natural antioxidant source. Moreover, the extraction method significantly influences antioxidant performance, with SFEO exhibiting stronger activity than SDEO. These results highlight SFEO's potential as a natural food preservative or functional skincare ingredient, supporting its further industrial development.

#### 3.5.2 Antibacterial activity

CEOs exhibit broad-spectrum antibacterial activity *in vitro*, although the extent of inhibition varies greatly depending on citrus species and extraction techniques (66, 67). To date, no studies have reported on the antibacterial properties of ZJPEO. In this study, antibacterial activity was assessed using the Oxford cup method and micro-broth dilution assays. As shown in Table 2 and Figure 6E, both SDEO and SFEO inhibited the growth of *Staphylococcus aureus (S. aureus)* and *Escherichia coli (E. coli)*. The inhibitory effect was significantly stronger against Gram-positive *S. aureus* than Gram-negative *E. coli* (*P*<*0.05*). This difference may be attributed to structural variations in the bacterial cell wall: Gram-negative bacteria possess a hydrophilic lipopolysaccharide (LPS) outer membrane that impedes the penetration of hydrophobic EOs components, whereas Gram-positive bacteria lack this barrier and are therefore more susceptible to EO-induced membrane disruption, cytoplasmic leakage, and metabolic inhibition (68). The extraction method also significantly affected antibacterial efficacy. Based on inhibition zone diameters, antibacterial potency followed the order SDEO < SFEO. MIC and MBC values (Table 3) further confirmed the stronger activity of SFEO. For *S. aureus*, SFEO exhibited MIC and MBC values of 0.125 μL/mL and 2 μL/mL, respectively, whereas SDEO exhibited 4 μL/mL and 16 μL/mL. Against *E. coli*, SFEO showed MIC and MBC values of 4 μL/mL and 32 μL/mL, compared with 16 μL/mL and 64 μL/mL for SDEO. It is worth noting that, as previously mentioned, the EOs may contain trace residual ethanol. According to literature reports, ethanol concentrations above 45% can affect bacterial growth and survival (69). However, the maximum concentration of EOs used in our experiments was 512 μL/mL, which corresponds to an ethanol content of approximately 2%, well below the minimum inhibitory concentration reported in the literature. Therefore, the antibacterial contribution from ethanol in the EOs of this study is indeed very limited. Collectively, SFEO demonstrated significantly stronger antibacterial activity against both bacterial strains than SDEO (*P*<*0.05*). This suggests that SFE more effectively extracts or preserves antimicrobial constituents compared to SD. From an industrial perspective, this finding holds significant practical value. Although SFE technology involves relatively high equipment investment, its green production advantages, including solvent recycling and residue-free extraction, contribute to higher resource efficiency in large-scale production systems (70, 71). The notable antibacterial activity of SFEO, coupled with the technical feasibility of large-scale continuous production and the trend toward equipment standardization, collectively enhances its potential for industrialization in applications such as personal care products and pharmaceuticals with antibacterial functions. Of course, in view of the complexity of the EOs composition, further clarification of its specific antimicrobial components and mechanism of action is still needed to support its industrial development and standardized production.

**Table 2:** Inhibition diameter of ZJPEO by different extraction methods (x ± s, *n* = 3).

**Strains**	**Inhibitory circle diameter (mm)**			
	**Control**	**SDEO**	**SFEO**	**Gentamicim**
*Escherichia coli* (*E.coli*)	–	14.28 ± 1.29^c^	23.02 ± 1.85^b^	30.78 ± 1.67^a^
*Staphylococcus aureus* (*S. aureus*)	–	22.39 ± 1.27^b^	29.44 ± 0.79^a^	29.72 ± 1.17^a^

Different lowercase letters in the same row represent significant differences (*P* < 0.05).

**Table 3:** MIC and MBC of ZJPEO with different extraction methods.

**Strains**	**SDEO**		**SFEO**	
	**MIC (**μ**L/mL)**	**MBC** **(**μ**L/mL)**	**MIC (**μ**L/mL)**	**MBC** **(**μ**L/mL)**
*Escherichia coli (E.coli)*	16	64	4	32
*Staphylococcus aureus (S. aureus)*	4	16	0.125	2

#### 3.5.3 Correlation analysis

**Figure 7:**
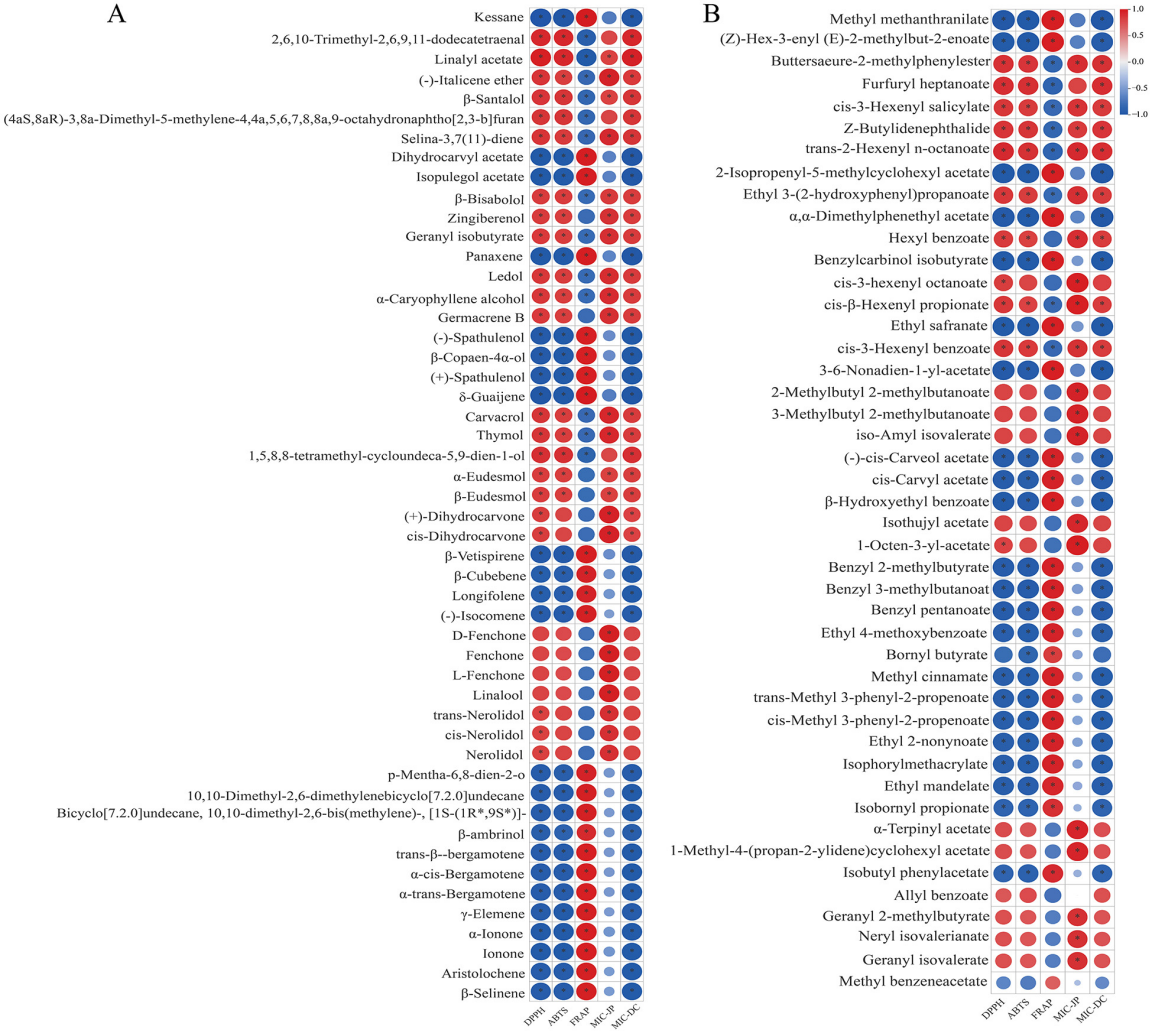
Correlation analysis between differential VOCs and antioxidant and antibacterial activities. **(A)** Terpenoids. **(B)** Esters. *Indicates a significant difference (*P* < *0.05*).

To explore the associations between bioactivities and differential VOCs, a Pearson correlation analysis was performed. The analysis included antioxidant activities (IC50 values used for ABTS and DPPH, FRAP expressed as total antioxidant capacity), antibacterial activities (MIC-JP for *S. aureus* and MIC-DC for *E. coli)*, and the top 50 terpenoids and esters (ranked by VIP values). As shown in Figure 7, 24 terpenoid components, including kessane, aristolochene, and longifolene, exhibited significant negative correlations with DPPH and ABTS, but significant positive correlations with FRAP total antioxidant capacity. Additionally, 22 esters, such as 3,6-nonadien-1-yl acetate, cis-methyl 3-phenyl-2-propenoate, and isobornyl propionate, showed significant negative correlations with DPPH. Twenty-three esters also displayed negative correlations with ABTS and positive correlations with FRAP. These discrepancies may arise from the structural complexity of VOCs and the distinct reaction mechanisms underlying the DPPH, ABTS, and FRAP assays (72). Regarding antibacterial activity, terpenoids such as thymol, linalool, and linalyl acetate, as well as esters including furfuryl heptanoate, cis-3-hexenyl salicylate and 1-octen-3-yl acetate, showed significant positive correlations with MIC-JP, suggesting strong contributions to the antibacterial activity against *S. aureus* (73, 74). These results agree with previous studies demonstrating the antimicrobial efficacy of thymol and linalool. Conversely, some VOCs exhibited negative correlations with antibacterial activity against *E. coli*. For instance, terpenoids such as aristolochene, γ-elemene and α-trans-bergamotene, and esters such as (*Z*)-hex-3-enyl (*E*)-2-methylbut-2-enoate, 2-isopropenyl-5-methylcyclohexyl acetate and ethyl mandelate showed significant negative correlations with MIC-DC. This study is the first to systematically elucidate the correlation between VOC composition in ZJPEO extracted by different methods and its bioactivities. These findings provide important insights for uncovering the mechanisms of active constituents and identifying novel bioactive compounds, offering valuable guidance for the development of new pharmaceuticals and functional cosmetics.

## 4 Conclusions

In this study, ZJPEO was extracted using two method, SD and SFE. The chemical composition of the resulting ZJPEO was comprehensively characterized using FTIR, E-nose, HS-GC-IMS, and HS-SPME-GC-MS. FTIR and E-nose analyses revealed notable compositional differences between SDEO and SFEO. HS-GC-IMS identified 63 VOCs, including terpenoids, alcohols, aldehydes, ketones, esters, and other compounds, with aldehydes, esters and terpenoids being the dominant classes. Based on VIP > 1, five VOCs (nonanal-*D*, octanal-*M*, mesityl oxide-*M*, (*E*)-2-hexenal, and ethyl formate-*M*) were identified as potential markers for differentiating ZJPEO obtained via distinct extraction methods. Complementarily, HS-SPME-GC-MS detected a total of 1,250 VOCs, revealing that terpenoids and esters are key contributors to the characteristic flavor and bioactivity profiles of ZJPEO. KEGG pathway enrichment analysis further indicated that sesquiterpene and triterpene biosynthesis pathways largely underpin these compositional differences. Flavor wheel analysis showed that SFEO exhibits more pronounced sweet, green and woody notes compared to SDEO. *In vitro* bioassays demonstrated that SFEO possesses strong antioxidant activity, effectively scavenging DPPH (IC50 = 2.31 mg/mL) and ABTS free radicals (IC50 = 3.71 mg/mL), with a total antioxidant capacity (FRAP) of 0.1701 mmol/g. Additionally, SFEO exhibited antibacterial activity against both *S. aureus* and *E. coli*. Pearson correlation analysis further revealed significant associations between specific VOCs and biological functions: compounds such as kessane, aristolochene, cis-methyl 3-phenyl-2-propenoate, and isobornyl propionate were correlated with antioxidant activity; thymol, linalool, and furfuryl heptanoate correlated with antibacterial activity against *S. aureus*; and aristolochene, γ-elemene, 2-isopropenyl-5-methylcyclohexyl acetate, and ethyl mandelate were correlated with antibacterial activity against *E. coli*.

In summary, this work provides the first comparative evaluation of the effects of SD and SFE on the chemical composition, flavor profile and bioactivities of ZJPEO. The findings demonstrate that extraction methods markedly influence the chemical profile of ZJPEO, thereby shaping its sensory and functional properties. SFEO, in particular, exhibited superior antioxidant and antibacterial activities, highlighting its strong potential as a natural preservative for pharmaceutical and cosmetic applications. Moreover, this study is the first to establish correlations between VOC composition and the bioactivities of ZJPEO. These insights lay a foundation for further mechanistic studies and bioactive compound discovery, promote the industrial application of ZJPEO and support the development of high-quality and high-value-added ZJPEO products, ultimately contributing to the sustainable and efficient utilization of Zhoupigan resources.

## Data Availability

The original contributions presented in the study are included in the article/Supplementary material, further inquiries can be directed to the corresponding author.
