# Supplementary material for: Unraveling the volatile profile and bioactivity of Zhoupigan (Citrus reticulata cv. Manau Gan) essential oil via HS-GC-IMS and HS-SPME-GC-MS: a comprehensive evaluation of different extraction techniques
Source: Front Nutr. 2026 Mar 3;13:1775469. doi: 10.3389/fnut.2026.1775469 (PMC12992264; doi:10.3389/fnut.2026.1775469)
Supplement: Supplementary file 1 [file Data_Sheet_1.docx]

**Supplementary material**

**Figure S1** Results of PCA analysis of electronic nose
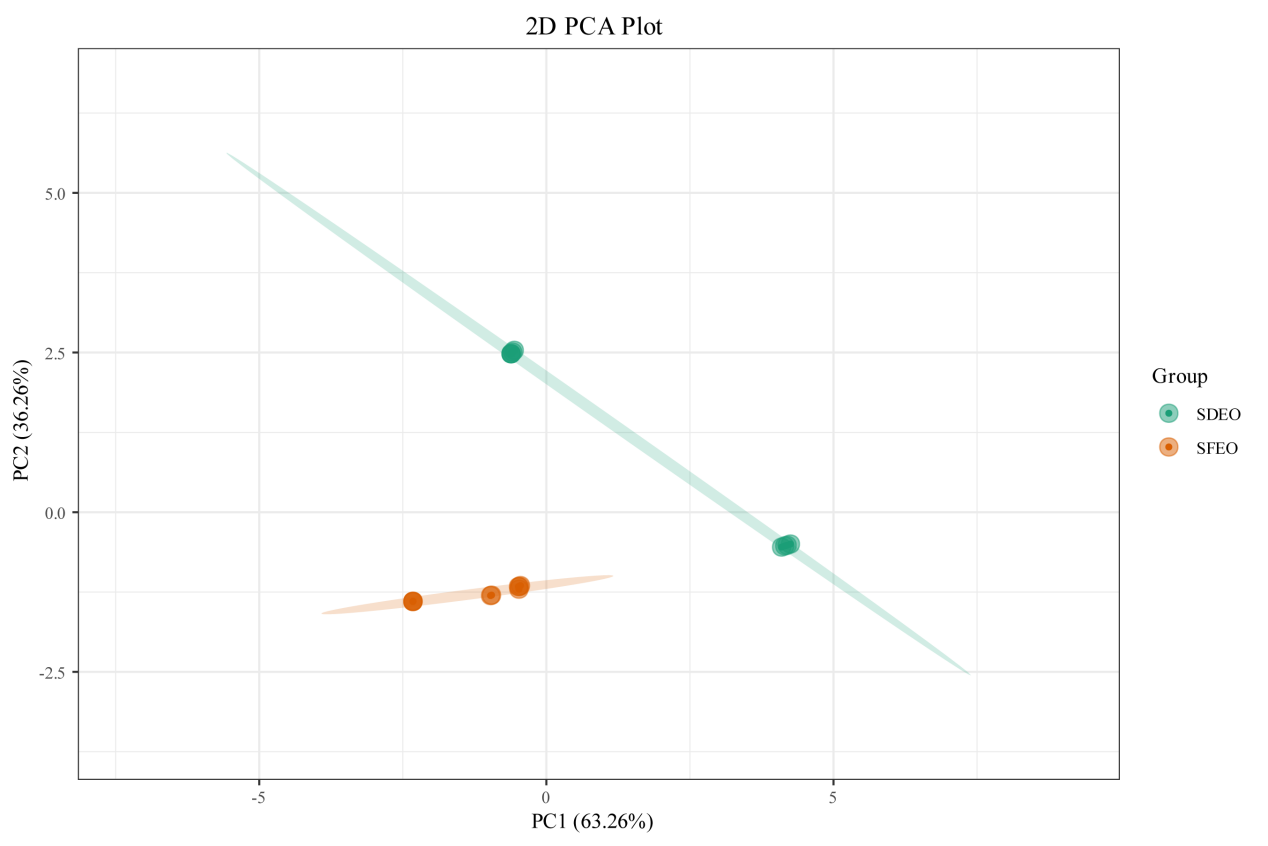


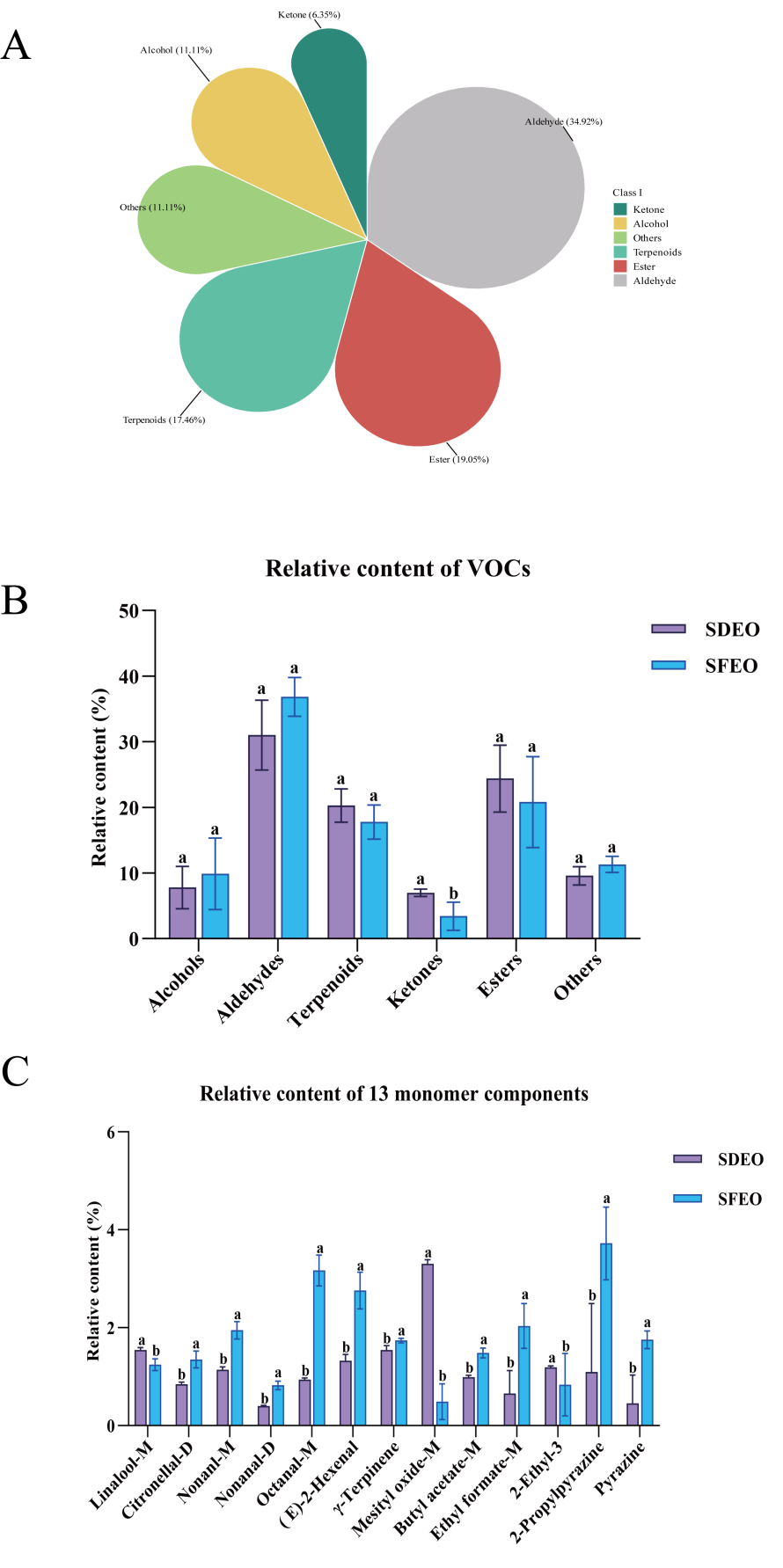


**Figure S2** Histogram of distribution and relative content of VOCs.

(A): VOCs rose pie chart. (B):Relative content of VOCs by category. (C): Relative content of 13 monomer components. Different lowercase letters indicate significant differences in the same index (*P<0.05*)


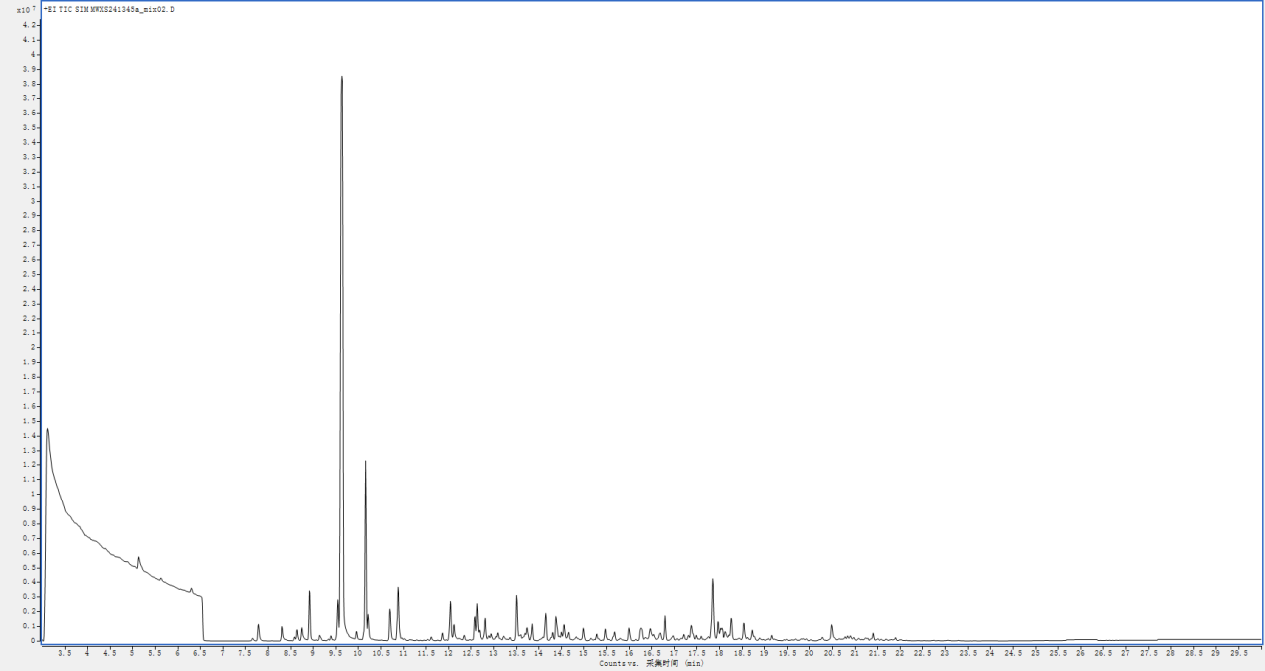


**Figure S3** Total Ion Flow Diagram
